# Machine Learning Strategies for Low-Cost Insole-Based Prediction of Center of Gravity during Gait in Healthy Males

**DOI:** 10.3390/s22093499

**Published:** 2022-05-04

**Authors:** Jose Moon, Dongjun Lee, Hyunwoo Jung, Ahnryul Choi, Joung Hwan Mun

**Affiliations:** 1 Department of Biomechatronic Engineering, College of Biotechnology and Bioengineering, Sungkyunkwan University, Suwon 16419, Korea; josemoon@skku.edu (J.M.); mouse87@skku.edu (D.L.); alex1130@skku.edu (H.J.); 2 Department of Biomedical Engineering, College of Medical Convergence, Catholic Kwandong University, Gangneung 25601, Korea

**Keywords:** center of gravity, balance, insole system, gait analysis, gait phase classification, feature engineering, bi-directional long short-term memory

## Abstract

Whole-body center of gravity (CG) movements in relation to the center of pressure (COP) offer insights into the balance control strategies of the human body. Existing CG measurement methods using expensive measurement equipment fixed in a laboratory environment are not intended for continuous monitoring. The development of wireless sensing technology makes it possible to expand the measurement in daily life. The insole system is a wearable device that can evaluate human balance ability by measuring pressure distribution on the ground. In this study, a novel protocol (data preparation and model training) for estimating the 3-axis CG trajectory from vertical plantar pressures was proposed and its performance was evaluated. Input and target data were obtained through gait experiments conducted on 15 adult and 15 elderly males using a self-made insole prototype and optical motion capture system. One gait cycle was divided into four semantic phases. Features specified for each phase were extracted and the CG trajectory was predicted using a bi-directional long short-term memory (Bi-LSTM) network. The performance of the proposed CG prediction model was evaluated by a comparative study with four prediction models having no gait phase segmentation. The CG trajectory calculated with the optoelectronic system was used as a golden standard. The relative root mean square error of the proposed model on the 3-axis of anterior/posterior, medial/lateral, and proximal/distal showed the best prediction performance, with 2.12%, 12.97%, and 12.47%. Biomechanical analysis of two healthy male groups was conducted. A statistically significant difference between CG trajectories of the two groups was shown in the proposed model. Large CG sway of the medial/lateral axis trajectory and CG fall of the proximal/distal axis trajectory is shown in the old group. The protocol proposed in this study is a basic step to have gait analysis in daily life. It is expected to be utilized as a key element for clinical applications.

## 1. Introduction

Dynamic stability of human movement is achieved through balance control in the contribution of visual, vestibular, and somatosensory inputs [1,2]. Coordinated movement of body segments that minimizes the displacement of the whole-body center of gravity (CG) is a motor mechanism that restores balance by controlling the imbalanced state where CG is located outside the base of support [3]. The relative motion of the CG to the base of support is usually described by relating them to the interactions of CG and the center of pressure (COP) [4,5]. A joint assessment of CG and COP provides a complete evaluation of dynamic balance control [5,6]. CG stability decreases with increasing age due to a decrease in postural control ability to restore balance. The reduced balance in the elderly is a major cause of falls during walking [7,8]. Therefore, gait analysis in elderly healthy can make early screening for degenerative gait disorders and gait imbalance [9].

There are two common methods used to calculate the CG trajectory: (1) kinematic method based on an optical motion capture system, and (2) kinetic method using a force platform [10,11]. With the kinematic method, a number of optical markers are attached to anatomical landmarks. Each segment’s CG is calculated based on three-dimensional trajectories and an anthropometric model. The entire body CG is calculated by a weighted-sum average of separate segment CGs. CG trajectory calculation with the kinetic method is based on Newton’s second law. The sum of external forces acting on a body is expressed as the product of the mass and acceleration of the body. CG trajectory is calculated using double integration of acceleration with a determination of integration constants and appropriate estimation of initial conditions. Although these two methods can calculate CG trajectory with high accuracy, they have limitations of requiring expensive laboratory equipment and highly skilled operator [12,13,14]. Particularly, there is an inconvenience for subjects with the kinematic method since they have to walk with multiple markers attached. With the kinetic method, restricted measurements are made for a few steps for a limited number of force platforms fixed on the floor.

Recent advances in wireless sensor technology have made it possible to measure daily life motions without space constraints using inexpensive sensor devices. Inertial measurement units (IMU) and smart insole systems are representative wearable sensor devices that have the advantage of being able to analyze human body motion using real-time temporal information [15]. IMU that measures acceleration, angular velocity, and magnetic field in three planes has been used in various studies to estimate CG trajectory using strapdown integration or inertial sensors network [16,17]. Due to the inconvenience of inertial sensors network that requires constructing a kinematic model by wearing IMUs on each body segment, strapdown integration of the signal obtained by the IMU attached to the surface of the fifth lumbar vertebra or the first sacrum close to the human body CG has been preferred [18]. IMU-based CG trajectory estimation methods have been continuously improved with sensor-fusion algorithms to compensate for the shortcomings of every single sensor. Nevertheless, IMU has shortcomings in the measurement of direct ground reaction force. An insole system is an unobtrusive wearable sensor for measuring ground reaction force and gait parameters [19]. Plantar pressure distribution measured by the insole system can reflect the dynamic balance control. It has been used in various fields of rehabilitation and exercise analysis [20,21,22]. In particular, inclination angles between COP and CG shows their relationship in a rate of angle change that describes the body’s dynamic control during locomotion. It can be used as quantitative information to evaluate the static or dynamic balance of the human body [23].

Despite the high correlation between the pressure information of an insole system and the CG, there is no direct equation to calculate the CG. This problem can be solved by using machine learning techniques. Machine learning is a powerful tool that can predict output values when a new input is given by iteratively training the target function to best map the relationship between input and output variables from multiple datasets [24]. Therefore, model performance can be improved by preparing input data to have a high correlation with output. As a representative example of data preparation, a method of temporally segmenting input data is used. Segmentation by signal and image characteristics can label the data and extract specialized features for each segment [25]. The availability of large amounts of data and the improvement of computational power have made it possible to perform high-accuracy predictions using a deep learning model composed of successive layers [26]. Among deep learning models, long short-term memory networks (LSTM) with a special structure in which the previous outputs are connected to data prediction of the present time [27]. It has been reported that LSTM has higher computational accuracy for time-series data than a feed-forward artificial neural network model [28]. The bi-directional LSTM (Bi-LSTM) model has a structure in which existing LSTM nodes are connected in forward and reverse directions. It is an improved model with better prediction performance for time series data by considering bi-directional information in the output layer [29]. For time-series bio-signal data, the deep learning model is effective in dealing with correlated signals and high deviation between subjects [30].

Although an insole system is a very efficient device for measuring human body balance, studies that use it to estimate the CG trajectory have not been reported yet. The purpose of this study was to present a new protocol for estimating the CG trajectory using pressure data obtained from a low-cost wireless wearable insole system. To this end, a wireless low-cost insole system composed of piezo-resistive sensors was fabricated and verified, and a CG estimation protocol using the insole pressure measure was proposed. A deep learning model following the proposed protocol was developed using data from young and old healthy male groups and its performance was evaluated.

## 2. Materials and Methods

### 2.1. Low-Cost Insole Prototype

**Figure 1:**
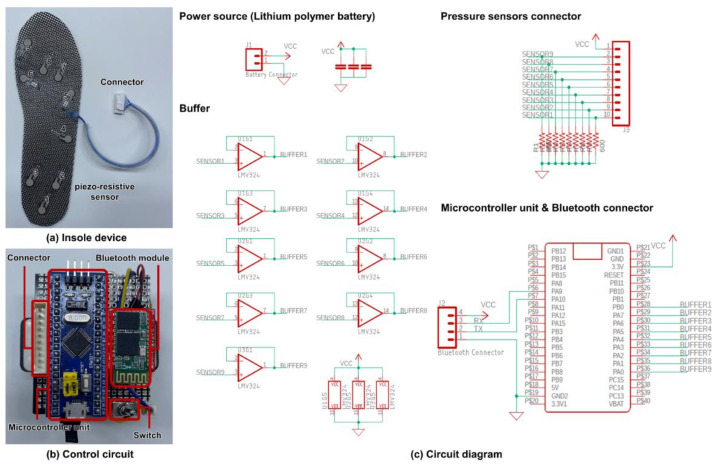
(**a**) Overview of the insole device and placement of the sensors; (**b**) control circuit of the insole system with connector, bluetooth module, microcontroller unit and switch; (**c**) circuit diagram of the custom-designed insole system.

The insole device manufactured for CG trajectory prediction consisted of an insole device with nine pressure sensors and a control circuit board that could convert the measured analog signal into a digital signal and transmit wireless data to the workstation (Figure 1). The pressure sensor attached to the insole device was a piezo-resistive sensor (Tekscan A301, Tekscan Inc., South Boston, MA, USA) in the form of a thin film. It has a characteristic that resistance changes according to the pressing force. The pressure sensor was attached to nine areas where the greatest pressure was generated during walking according to the anatomical structure of the foot [31]. It was manufactured in three sizes in consideration of subjects’ foot sizes (250 mm, 260 mm, and 270 mm). The control circuit board consisted of a microcontroller unit (STM32F103C8, STMicroelectronics, Geneva, Switzerland) for converting the measured analog signal to digital signal, a battery (3.7 V 2000 mhA) for driving the device, a Bluetooth 2.0v module for wireless data transmission, and a number of elements. A resistor distribution circuit was constructed to continuously measure sensor resistance that could change according to the magnitude of the pressure. The measured voltage was converted into a digital signal in the microcontroller unit. The converted digital signal was wirelessly transmitted at a rate of 100 samples/s through the Bluetooth module. The pressure measurement value of the manufactured insole was acquired using the LabVIEW program (LabVIEW ver. 20.0, National Instruments Corp, Austin, TX, USA).

Calibration was performed to convert a sensor signal measured in voltage unit into a pressure value. Calibrations for individual sensors were performed according to characteristics of the piezo-resistive sensor with different resistance values for each sensor at the same pressure [32]. Calibration was performed by attaching a zig device that could apply a vertical force to a digital push-pull gauge meter (DTG-100, DIGITECHCO. Ltd., Osaka, Japan) with a built-in load cell. The sensor was placed on a digital push-pull gauge meter fixed plate. A linearly increasing pressure was applied to measure the sensor voltage value against the pressure. For each sensor, calibration was performed three times. Measured values were established in the form of an exponential function using Matlab curve fitting toolbox version R2018b (Mathworks, Natick, MA, USA).

### 2.2. Subjects and Experimental Protocols

**Figure 2:**
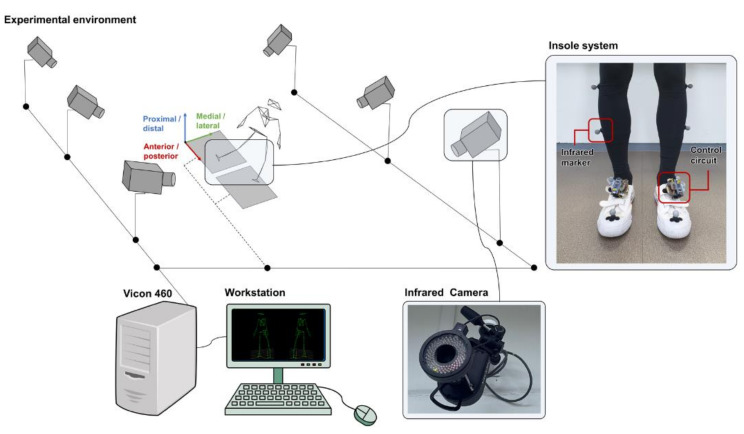
Overview of the experimental environment: infrared cameras receive signals of infrared markers attached to the human body and custom designed insole system placed under the foot.

For the experimental subjects of this study, 15 healthy adult males (age: 25.67 ± 2.01 years, height: 172.28 ± 6.71 cm, weight: 69.59 ± 6.13 kg) and 15 healthy elderly males (age: 77.33 ± 5.80 years, height: 168.24 ± 5.59 cm, weight: 65.76 ± 5.86 kg) over 60 years of age without history of musculoskeletal disorders were recruited. All experiments were approved by the local ethics committee (IRB No. 2018AN0297). They were conducted in the Biomedical Engineering Lab of Sungkyunkwan University in accordance with the experimental protocol. Written informed consent was obtained from all participants before the experiment. Each subject wore shoes of same design with flat sole. The shoe’s original insole was also removed to have flat and rigid contact. Optical markers were attached to 35 anatomical boundaries according to the modified Helen-Hayes marker set [33]. Six MCAM2 cameras (VICON, Oxford Metrics, Oxford, UK) driven at 120 Hz were used for CG trajectory calculation based on the kinematic method (Figure 2). All subjects performed warm-up exercises before participating in the experiment and they were allowed sufficient practice walking to adapt to the experimental environment. This period allowed to make similar insole temperature to human body, limiting drift and associated measurement errors [34]. As for the walking speed, subjects walked at a comfortable pace that they would normally walk [35]. Average walking speed of subjects was 1.41 ± 0.05 m/s. Participants performed level-ground walking from a start to a finish line (straight 8 m walkway). Each subject performed at least seven trials. The camera system and the insole system were manually synchronized based on major gait events such as heel strike and toe-off [28].

### 2.3. CG Prediction Protocol

**Figure 3:**
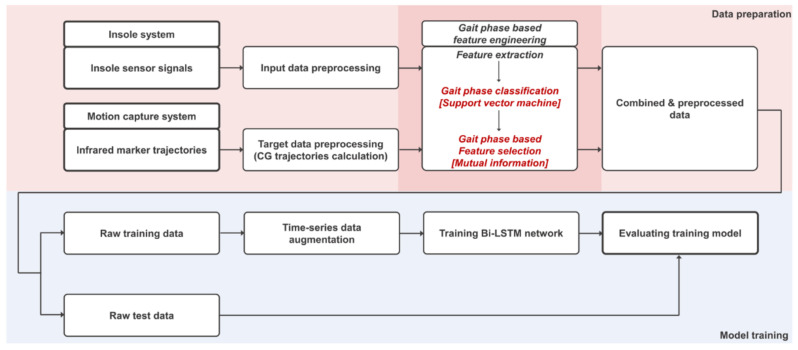
Overall architecture of the proposed protocol for CG prediction using an insole system.

The protocol proposed in this study consisted of a data preparation process and a model training process. The overall flow is shown in Figure 3. Input and target data preprocessing, gait phase-based feature engineering, data augmentation, Bi-LSTM network model, comparative study, and statistical evaluation are the processes. Each process is described in detail in the following section.

#### 2.3.1. Input and Target Data Preprocessing

Data obtained from the nine pressure sensors attached to the left and right feet, respectively, were used as input data. The 3D trajectory of the infrared marker obtained from the motion capture system was used as the target data of the model. For pressure sensor signals and marker trajectory data obtained during walking, high-frequency noise was removed by applying a 4th-order butter-worth lowpass filter. Cut-off frequencies of 7 Hz and 10 Hz were applied through residual analysis, respectively [35]. For both input and target data, time series data were extracted based on the heel strike to heel strike of the right foot of a gait cycle. Data were normalized to 100 frames and the measured pressure values were normalized to the subject’s weight [36].

The trajectory of the CG of the human body was calculated using the Vicon Plug-in-Gait model (kinematic method). The calculated CG trajectory data was converted to a local coordinate system with the middle point of double support as the origin during walking. The anterior/posterior direction (x-axis) of the local coordinate system was selected based on the walking direction. The proximal/distal direction was the same as the global z-axis. The medial/lateral direction (y-axis) was set as a cross product of the proximal/distal direction (z-axis) and the anterior/posterior direction. The calculated CG was normalized to the subject’s leg length to remove the effect of height difference [37].

#### 2.3.2. Gait Phase-Based Feature Engineering

**Figure 4:**
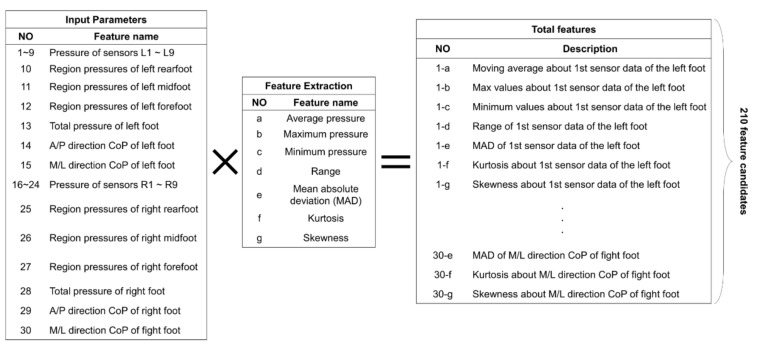
Engineered features. A total of 210 feature candidates were calculated by compounding input parameters and feature extraction features within five windows (A/P: anterior/posterior, M/L: medial/lateral).

Feature engineering is an important process for determining the accuracy of a model. It is a step in which the learning model selects a feature that can perform accurate prediction among several feature candidates [38]. As a first step, gait analysis was performed using a pressure sensor that calculated 30 input parameters based on previous studies [39,40] and extracted 210 features by applying a sliding window of 5 frame sizes to 7 time-domain features (Average, Maximal, Minimum, Range, Mean absolute deviation, Kurtosis, Skewness) (Figure 4). A total of 240 feature candidates were composed by adding raw data (30 features) corresponding to the last value of each window along with extracted features.

**Figure 5:**
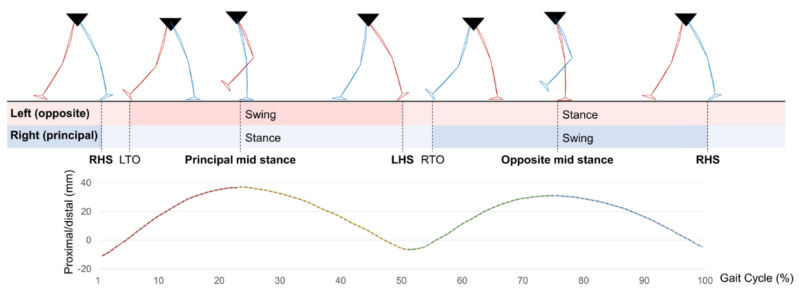
Description of four gait phases. A gait cycle is separated into four phases based on the proximal/distal movement of the CG (RHS: Right heel strike, LTO: Left toe−off, LHS: Left heel strike, RTO: Right toe−off).

Gait is a movement in which both feet cross each other’s stance phase and swing phase. One gait cycle is defined as the heel strike of the principal leg to the next heel strike. It is used as a reference section for intra-subject or inter-subject analysis in human kinematics studies using gait experiments. In particular, it is possible to derive a subdivided characteristic through a phase divided into several gait events in one gait cycle [41]. In this study, gait phase segmentation was performed to divide the gait cycle into four phases [42]: (1) principal foot heel strike to mid-stance, (2) principal foot mid-stance to opposite foot heel strike, (3) opposite foot heel strike to mid-stance, and (4) opposite foot mid-stance to principal foot heel strike based on the heel strike of the stance leg and the mid-stance event (Figure 5). Whenever the section is changed within the gait cycle, a pole where the CG vertical trajectory is converted appears. At this time, the conversion between the gravitational potential energy and the kinetic energy for gait efficiency and posture control occurs [43]. Therefore, the proposed gait phase segment enables the construction of a predictive model suitable for changes in mechanical properties of CG. In addition, since single support and double support are mixed in each phase (Phase 1, 3: single and double support; Phase 2, 4: single support only), the performance of the prediction model can be improved by selecting input feature points suitable for the phase. Heel strike and mid-stance events were detected using a kinematic model calculated in the motion capture system. For real applications where kinematic model calculation is impossible, an auto-classification model that classifies gait phases by insole pressure input can be developed.

The SVM used to classify the gait phase is one of the machine learning methods that can select the hyperplane that best distinguishes the input data set [44]. SVM is known to be quite beneficial in gait analysis because of generalization ability even for small amounts of data [45]. In this study, SVM was trained with 240 feature candidates as input and gait phase labels as output. A total of 30 subjects (15 young, 15 old, 3 trials for each subject) were used for cross-validation (CV). CV was performed five times. For each CV, 26 subjects (13 young, 13 old) were used for training, 2 subjects (1 young, 1 old) were used for validation, and 2 subjects (1 young, 1 old) were used for the test data set. Candidates of SVM parameters used for grid search parameter optimization were: ‘C’, {0.1, 1, 100, 1000}; ‘kernel’, {‘rbf’, ‘poly’, ‘sigmoid’, ‘linear’}; ‘degree’, {1, 2, 3, 4, 5, 6}; and ‘gamma’, {1, 0.1, 0.01, 0.001, 0.0001}. Regarding hyperparameters obtained through optimization, ‘C’ = 1, ‘kernel’ = ‘rbf’, and ‘gamma’ = 0.1 were used.

Feature selection can reduce the input dimension of the predictive model to a combination of highly relevant features, thereby preventing the increase in computational cost of the model due to unnecessary features and improving the predictive performance of the model. As a feature selection method, mutual information (MI) was used. MI represents the amount of information indicating the relationship between two random variables [46]. Feature selection using MI can calculate the MI of the target and all features to derive feature ranking. The equation used for MI calculation is shown as follows:

(1) $$I\left(X,Y\right)=\underset{x\in X,\;y\in Y}{\sum }p\left(x,y\right)\cdot \log\left(\frac{p\left(x,y\right)}{p\left(x\right)p\left(y\right)}\right)$$

**Figure 6:**
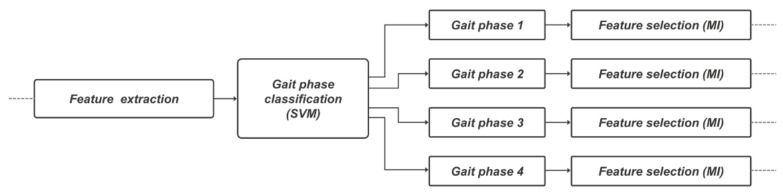
Details of gait phase-based feature engineering process using support vector machine and mutual information.

Here, $I\left(X,Y\right)$ represents the MI for two variables *X* and *Y*. $p\left(x,y\right)$ denotes the joint probability distribution function of a and b, and $p\left(x\right)$ and $p\left(y\right)$ denote the marginal probability distribution function. In each classified gait phase, MI with 240 feature candidates was derived by targeting (anterior/posterior, medial/lateral, and proximal/distal) CG trajectories in the 3-axis direction. After scaling MI values calculated for each direction to 0~1, the average value was derived and scored and features representing each phase were selected. Optimization was performed to select the optimal number of features using the relative root mean square error of the CG trajectory calculated through the support vector regressor (SVR) as the objective function. The flowchart of gait phase-based feature engineering is shown in Figure 6.

#### 2.3.3. Data Augmentation

Data augmentation can prevent overfitting of the model and increase robustness by increasing the amount of training data by generating pair samples. Data augmentation can change data in magnitude and time domains. The magnitude data augmentation technique changes the original signal intensity of time series data to confuse label characteristics, thereby reducing model accuracy [47,48]. Therefore, in this study, three types of time-domain augmentation (jittering, time-warping, and pooling) techniques were applied [47,49]. Time warping is a method of changing temporal properties of samples by distorting the time interval between samples. Time warping based on a random smooth warping curve generated by cubic spline with four knots at random magnitudes (µ = 1, σ = 0.2) [47]. Jittering is a method to enhance the robustness of the training model by adding white Gaussian noise to the training data. Random noise from a Gaussian distribution with a mean µ = 0 and a standard deviation σ = 0.03 is added to the original time series [47]. Pooling is a method to reduce the resolution without changing the length of time series data. by averaging a pooling window. We use a window of size 3 [47].

Twenty-six subject data (13 healthy young males, 13 healthy old males) were augmented and used for training. The size of the training data was 26 subjects × 3 trials × 100 frames × 4 folds augmentation (1 raw signal, 3 augmented signals) = 31,200, 2 subjects (1 healthy young male, 1 healthy old male, 3 trials and 100 frames for each subject) were used for validation and 2 subjects (1 healthy young male, 1 healthy old male, 3 trials and 100 frames for each subject) were used for the test data set.

#### 2.3.4. Bi-LSTM Network Model

**Figure 7:**
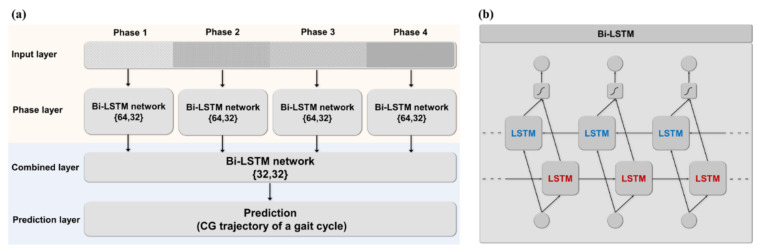
Details of prediction model using Bi-LSTM network. (**a**) prediction model structure (**b**) architecture of Bi-LSTM network.

To estimate the sequential CG trajectory, Bi-LSTM deep learning model specialized for time-series data prediction was used in this study. Bi-LSTM model is a class of LSTM model that can learn information from multiple time frames (t – n, …, t − 1), which is the limit of RNN in which only the information of the previous frame (t − 1) is used for time frame (t) prediction [29]. Bi-LSTM has a structure in which LSTM nodes are connected in forward and reverse directions. Prediction is performed using subsequent time frame information in addition to previous time frame information. The core prediction structure of the Bi-LSTM model is shown in Figure 7a. The internal structure of the Bi-LSTM cell is shown in Figure 7b. In the part shaded in red as shown in Figure 7a, learning is carried out in each gait phase. Input passes through independent Bi-LSTM layers {64, 32} with features selected appropriately for each phase. In the part shaded in blue shown in Figure 7a, four arrays derived through the phase layer are combined into one to pass through the Bi-LSTM layer {32, 32}. Data passed through the combined layer are used to predict the 3-axis CG trajectory through the prediction layer. The array extracted through the phase layer and the combined layer contains local features suitable for phase prediction. Prediction layer is designed to perform 3-axis CG trajectory prediction.

Input and output data were normalized to 0 and 1 for model optimization. The parameters used for hyperparameter optimization are ‘batch size’: {5, 10, 50}, ‘initial learning rate’: {0.1, 0.01, 0.001}, ‘optimizer’: {‘adam’, ‘adamax’, ‘rmsprop’}, ‘batch size’: 10, ‘initial learning rate’: 0.001, ‘optimizer’: ‘adam’ were used as hyperparameters obtained through optimization. In addition to the optimized parameters, max epoch was 1000 in the algorithm, sigmoid is used as the activation function, and the number of hidden units in each layer is shown in Figure 7. For the model configuration, Keras from TensorFlow was used. In order to limit overfitting, in addition to the already mentioned parameters, the callback functions ‘ReduceLROnPlateau’ and ‘EarlyStopping’ provided by Keras are used [50]. ‘ReduceLROnPlateau’ is a callback function that induces model improvement by adjusting the learning rate when there is no model improvement. ‘EarlyStopping’ is a callback function that terminates training in advance during training if there is no improvement in validation loss within max epoch. The model implementation used Python 3.7 version using a RTX 2080Ti GPU (4352 CUDA cores, 1665 MHz base clock speed, and 11 GB RAM).

### 2.4. Comparative Study

**Figure 8:**
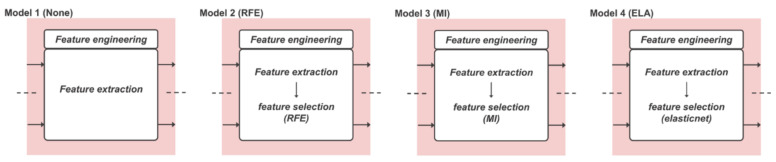
The feature engineering sequence of comparative models is different from the Proposed model.

Four comparative models were designed to evaluate the performance of the gait phase-based CG trajectory prediction model proposed in this study. Feature engineering process, which is different between the comparative models and the proposed model, is shown in Figure 8. Unlike the proposed models, feature engineering processes of the comparative models did not use the segmented gait phases, input features from a whole gait cycle were used. Model 1 (None) predicted the CG trajectory using data without feature selection as the Bi-LSTM network input. In Model 2 (recursive feature elimination, RFE), Model 3 (mutual information, MI), and Model 4 (elastic net, ELA), CG trajectories were predicted using features selected by applying different feature selection methods without gait phase discrimination. Models 2–4 representing three categories of feature selection methods (wrapper method, filtering method, embedded method) [51] were selected. Wrapper methods use machine learning to select features according to the performance of the model. RFE is a representative wrapper method. By calculating a feature-specific importance score using machine learning, features with low scores are removed from the subset. This process is repeated until the set number of remaining features is reached. The filtering method is a method that can select features based on the statistical significance between each input feature and target using a statistical measurement method without using a predictive model. MI is a representative method (discussed in Section 2.3.2). The embedded method is a mixture of wrapping and filtering methods. It is configured to perform feature selection during the training process of the predictive model. As a representative method, elastic net can group variables with correlation among variables from feature candidates. With L1 regularization (lasso regression) and L2 regularization (ridge regression), elastic net performs automatic variable selection and continuous contraction at the same time and can select a group of correlated variables. Elastic net is a method of including the entire group to which the variable that has a strong correlation with the dependent variable belongs in model building.

### 2.5. Statistical Evaluation

The performance of the SVM-based gait phase classification model was evaluated with precision, recall, and f1 score [52]. Correlation coefficient, root mean square error (RMSE), and relative RMSE (rRMSE) [28] were used to evaluate the performance of the CG trajectory calculated by the kinematic method and five predictive models. Analysis of variance (ANOVA) was performed for statistical comparison of the error rate between each model. Tukey test was used as a post hoc test. Significance levels were set at *p* < 0.05 and *p* < 0.01. Performance evaluation was calculated with Python 3.7. All statistical analyses were performed using the PASW Statistics version 18 (SPSS Inc., Chicago, IL, USA).

## 3. Results

### 3.1. Sensor Calibration and Validation

**Figure 9:**
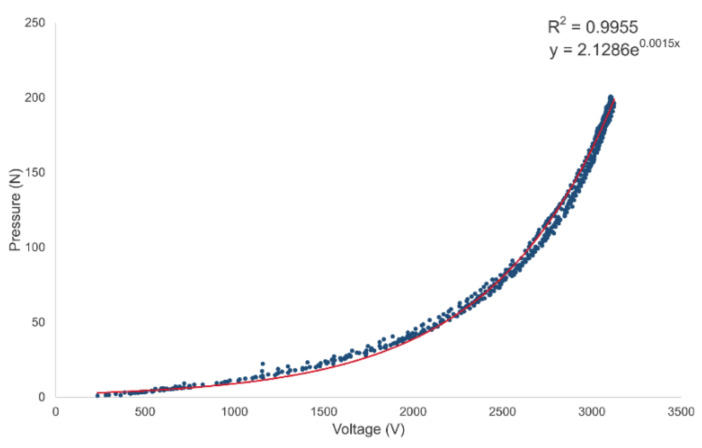
Sensor calibration result. Sensor output-pressure versus voltage.

Figure 9 shows the correlation between the voltage value and the pressure value for the representative sensor measured during the calibration process. Two measured values showed a non-linear relationship. The relationship was established using an exponential function. The relational formula established for each sensor showed an average correlation coefficient of 0.98 (±0.05) and a root mean square error (RMSE) of 4.14 (±1.49) N when compared with the actual measured pressure value. All nine pressure sensors showed a similar trend.

### 3.2. Input Feature Selection

**Figure 10:**
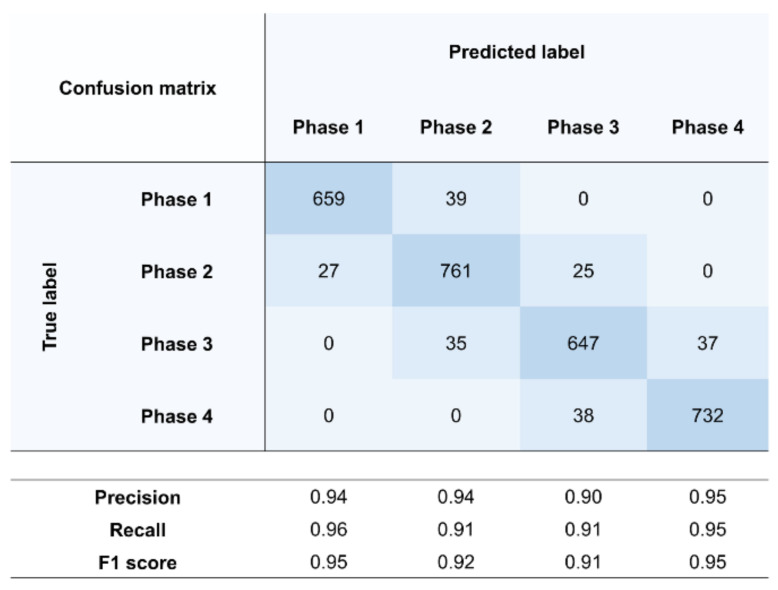
Confusion matrix, precision, recall, and f1 score of results of gait phase classification.

Figure 10 shows a confusion matrix for gait phase classification. A test data set of 3000 frames was constructed using 30 trial data sets (5 CV × 3 trials × 2 subjects (young 1, old 1)) consisting of a gait cycle of 100 frames. Among these three thousand data, 698, 813, 719, and 770 frames of data belonged to phases 1, 2, 3, and 4, respectively. Misclassification occurred only when the phase was changed. 

**Figure 11:**
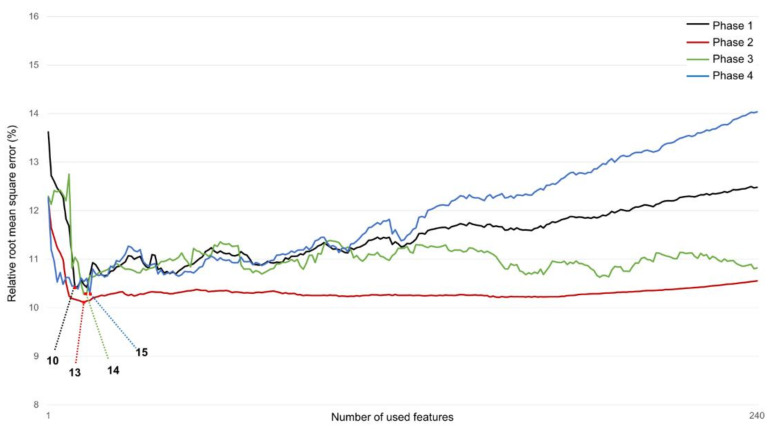
Relative root mean square error (%) computed during feature number optimization to use variables as input of Bi-LSTM network.

Figure 11 shows the error rate derived from the optimization process for selecting the optimal number of features for each phase. In Phase 1, when 10 features were used, an error of about 10.41% was shown. Thereafter, as the number of features increased, the error also showed a tendency to increase. In Phase 2, 13 features converged to an error rate of 10.11%. An approximate error was calculated even when features were added. Phases 3 and 4 had the smallest errors when 14 and 15 features were used, respectively. Accordingly, the optimal number of features according to each phase was selected and used as the Bi-LSTM model input.

**Figure 12:**
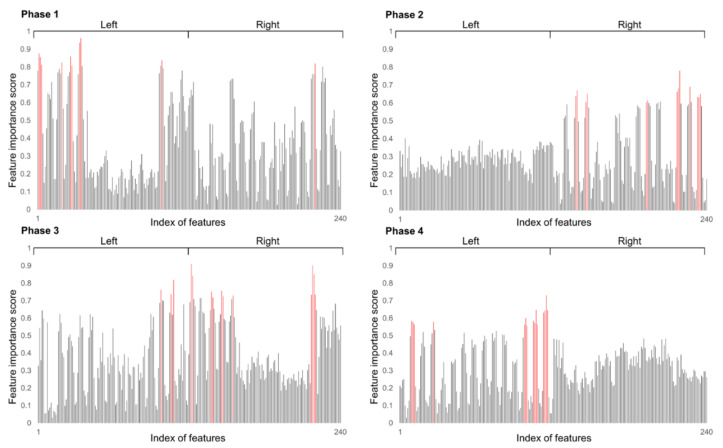
Feature selection result. Selected features from 240 candidates are colored red.

Input feature selection results for each phase are shown in Figure 12 and Table 1. Figure 12 shows the feature importance score based on MI feature selection for 240 feature candidates. In each phase graph, the left 120 features mean feature candidates calculated from the left foot and the right 120 features mean feature candidates calculated from the right foot. In Phase 1, all features except one from the right foot were derived from left foot features. In the case of Phase 2, all features were selected from the right foot. Among the features of the right foot, six front foot signals and four COP components were selected. In Phase 3, features on the left and right foot were evenly selected. The front area of the right foot (sixth, seventh, eighth sensors and front foot) components accounted for about 60% of the selected features. In the case of Phase 4, all 15 selected features were selected from the left foot. Among them, the COP component occupied the most weight. Features finally selected for each phase are shown in Table 1.

### 3.3. Prediction Performance of CG Trajectory

**Figure 13:**
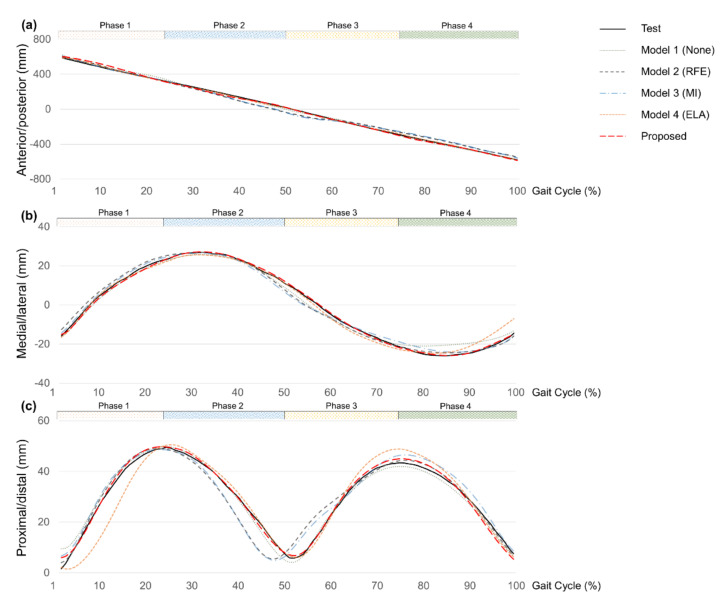
CG trajectories of measurement and prediction (None, RFE, MI, ELA, Proposed). (**a**) Anterior/posterior direction; (**b**) medial/lateral direction; (**c**) proximal/distal direction.

Figure 13 shows estimated CG trajectories of three directions calculated by the kinematic method. The predicted trajectories are from four comparative models and the proposed prediction model during one cycle of gait of a representative subject. 

**Figure 14:**
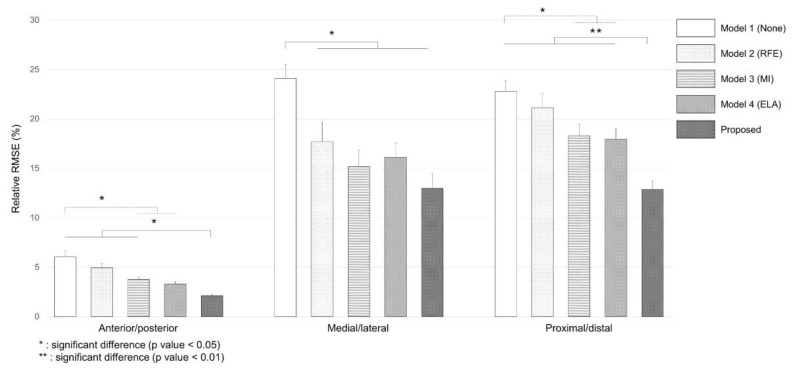
Average relative root mean square errors of CG prediction for five different models.

Figure 14 shows relative RMSE comparison results for each model in each direction for the total subject. In the anterior/posterior direction, there was a significant (*p* < 0.01) difference in the rRMSE value with the remaining models compared to the Proposed model. The rRMSE of the Proposed model was 2.12 ± 0.13%, showing the least error. In the medial/lateral direction, there was a significant (*p* < 0.01) difference in the rRMSE value with the remaining models showing reduced error compared to None. The rRMSE was 24.08 ± 1.40% for None and 12.97 ± 1.48% for the Proposed model, showing the largest difference. In the proximal/distal direction, the rRMSE was 12.47 ± 1.85% for the Proposed model and 17.96 ± 1.06% or more for the remaining models, showing a significant difference (*p* < 0.01). The rRMSE was 22.77 ± 1.08% for None, showing a difference between MI and ELA at *p* < 0.01 level. These results revealed that the Proposed model, which performed all proposed protocols, showed the lowest error rate in the prediction of three directions.

Table 2 shows the prediction results of young and old groups by model. The CG trajectories prediction accuracy for each group of subjects in each model was compared based on three performance criteria: correlation coefficient, RMSE, and rRMSE. Representatively, with the Proposed model, correlation coefficients on anterior/posterior, medial/lateral, and proximal/distal axes for the young subject group were 0.99 (0.99–0.99), 0.92 (0.98–0.75), and 0.92 (0.98–0.60) with RMSE of 26.73 ± 2.92 mm, 8.72 ± 1.68 mm, and 6.12 ± 0.72 mm, respectively, (rRMSE: 2.13 ± 0.21%, 14.24 ± 1.72%, and 14.01 ± 1.09%). The Proposed model showed improved prediction results in most directions. As a result of young subject CG prediction, in the anterior/posterior direction of rRMSE, None and the RFE model showed a significant difference with a significance level of 1% compared to the Proposed model. In the medial/lateral direction, compared to the Proposed model, None and the RFE model showed a significant difference at *p* < 0.01 and *p* < 0.05, respectively. In the proximal/distal direction, all models showed a difference from the Proposed model at the significance level of 1%. In the case of old subjects, only the Proposed model and None showed a difference (*p* < 0.01) in the anterior/posterior direction, and only the Proposed model and the MI model showed a difference at *p* < 0.05 in the medial/lateral direction. In the case of the old subjects, the None model and the RFE model showed a difference in *p* < 0.01 compared to the Proposed model in the anterior/posterior direction, and the MI model showed a difference in *p* < 0.05. The None model showed a difference in significance level of 5% in the medial/lateral direction and 1% in the proximal/distal direction compared to the Proposed model.

## 4. Discussion

The existing CG measurement method based on a motion analysis system has a major limitation in requiring expensive apparatuses with limited measurement volumes, as discussed in the Introduction. In this study, with characteristic coincidence between gait phase events and the CG trajectory peaks [53], a gait-phase-based input segmentation and feature engineering method was proposed. Our proposed method was able to select optimal input features for gait phase segments. The entire CG trajectory prediction protocol was designed by using the data augmentation technique that could improve the quality and quantity of training data and deep learning algorithm. CG trajectory was predicted from gait pressure data of young and old group subjects measured using a self-made insole system. CG trajectory prediction performance was validated through statistical analysis by comparing with CG prediction models having the traditional feature selection method with an unsegmented gait cycle.

The insole system is one of the state-of-the-art technologies used in gait analysis. In this study, an insole system composed of nine piezoresistive sensors was manufactured and pressure distribution was measured with gait experiments. Pressure sensors were positioned at points where the pressure was concentrated in gait using a heat map measured with a commercial Pedar-X mobile system [31,54]. Sensors were distributed at three zones (rear, mid, and forefoot) [55] and sequential gait events (heel strike, mid stance, and push-off) of foot-ground interactions could be accurately measured. For the healthy male group, CG trajectory prediction results of the Proposed model using the fabricated insole system were satisfactory with anterior/posterior of 0.99, medial/lateral of 0.92, and proximal/distal of 0.92 correlations (Table 2) It is difficult to interpret the exact cause of the high prediction result due to the structure of the machine learning model where the intermediate learning process is an unknown black box [56]. Still, the feature engineering step to select gait phase-dependent features and the data augmentation step to create new data patterns that have not been acquired are considered factors leading to the high prediction accuracy. However, the number and location of the sensors in this study cannot be guaranteed as an optimal arrangement for CG estimation. A more improved CG estimation can be accomplished through hardware-oriented research studies to find the optimal sensor location and number.

Prediction results showed that the Proposed model with gait phase segmentation had the most accurate CG trajectory in all three planes (Figure 14). The training method by dividing the entire input data into several temporal segments is a method mainly used in pattern recognition and classification, which combines segments with an independent LSTM cell to extract features that can accurately distinguish a class, thus increasing model classification accuracy [57]. A typical segmentation method using a sliding window with a fixed frame is generally used. However, it has been revealed as an ineffective approach for training irregular transitions that occur in human body motions with different characteristics [58]. Since there was the requirement for semantic segmentation by motions, mid-stance and heel strike events were used as breakpoints dividing one gait cycle in this study. Mid-stance is the moment when the hip and ankle joint center of the stance leg and the CG trajectory is perpendicular to the ground. At the moment, the gravity potential energy converted from kinetic energy has the highest amount. The energy flow is reversed as the CG moves into the front of the ground contact point [43]. Conversion to the kinetic energy moves the human body forward along with vertical CG fall. The heel strike acts as a mechanism that stops the CG fall of collapsing balance and turning point of gathering gravitational potential energy. Statistically significant rRMSE of the proposed method (Figure 12) showed that input segmentation also had an effect on improving the prediction accuracy of the regression model, thus boosting the inherent sliding window-based featuring of the deep learning model. More segmented data segmentation would be required if patient data showing various gait patterns are added. An in-depth model training can then be performed.

Biomechanical analysis should be able to quantitatively measure an individual’s motor performance and represent significant differences between different subject groups [59]. It is possible to diagnose and evaluate balance maintenance ability using various parameters derived from CG, such as XCoM and CoMv [60]. As a study to evaluate the possibility of biomechanical analysis using the predictive model, young and old group differences were analyzed using the peak to valley range (PV range) [61]. PV range was quantified by subtracting values from the peak and valley on the CG trajectory. It was used to evaluate differences in medial/lateral and proximal/distal directions according to age (Table 3). When average PV ranges of the young and old groups were calculated using the kinematic method, they were significantly (*p* < 0.05) higher in the old group in both medial/lateral and proximal/distal directions. The proposed prediction model also showed statistically significant differences (*p* < 0.05) in medial/lateral and proximal/distal directions in the same manner as the kinematic method showed. The large sway of the medial/lateral axis trajectory and the fall of the proximal/distal axis trajectory shown in old group gait typical patterns have been reported in previous studies [53,60,61,62], indicating the low balance control ability. In terms of the motor control mechanism of gait, a decrease in hip abductor muscle strength with aging can reduce the medial acceleration of CG, resulting in greater medial/lateral CG displacement. A decrease in active braking of the plantar flexors group for vertical CG fall can result in an increase in proximal/distal CG displacement. The equaling statistical difference of the predictive model from the kinematic method means that the insole-based CG predictive model can provide quantitative individual measures and well distinguish different groups.

There are several limitations to overcome before applying the proposed wireless insole system-based CG prediction model in daily life. In this study, the model was trained using limited conditions of a gait experiment targeting healthy elderly and normal young adults. However, it is known that gait speed, disease status, age, and gender differences are factors affecting gait characteristics [63]. The purpose of this study was to present a protocol for estimating gait CG using pressure data of the low-cost insole system for the first time. A robust model validation study comprising various subject datasets presented above is needed so that the prediction model can be used for clinical or rehabilitation monitoring in the future. In addition, the protocol presented in this study focused on generating optimal training input through data preparation steps so there is a limitation in that the data training model is limited to the Bi-LSTM model. Various verified deep learning architectures exist, including convolutional neural networks (CNN). A future study is needed to optimize the training and prediction process by differing various deep learning models combined with the proposed data preparation method. Additionally, an empirical study with a longer period of sensor measurement is required. A potential concern with plantar pressure systems is their drift over time, which will be important when the systems are used in real-life settings for long periods of time.

## 5. Conclusions

In this study, a deep learning model-based protocol for a low-cost insole-based CG trajectory estimation was proposed. The gait phase segmentation process allows us to effectively improve the prediction accuracy in the presented protocol. It contributed to the improvement of the learning efficiency by selecting optimized features according to the gait phase. When the prediction accuracy was compared with those of four comparative models, in which the gait cycle segmentation was not performed, the Proposed model showed the highest performance (2.12% for anterior/posterior, 12.97% for medial/lateral, and 12.47% rRMSE for proximal/distal). This study showed that the proposed deep learning architecture that independently trained four subdivided gait phase segments could effectively estimate CG trajectories. The protocol proposed in this study is a basic study for gait analysis in daily life and is expected to be utilized as a core element of a rehabilitation monitoring system for postural control ability evaluation and balance recovery of the elderly.

## Figures and Tables

**Table 1:** Selected features in each phase using mutual information.

Phase	Description
Phase 1	Max values about 6th sensor data of the left foot; Moving average about 6th sensor data of the left foot; Moving average about sum of total signals from the left foot; Max values about 7th sensor data of the left foot; Max values about sum of total signals from the left foot; Max values about front foot; signals of the left foot; Minimum values about 8th sensor data of the left foot; Minimum values about front foot; signals of the right foot; Minimum values about sum of total signals from the left foot; Minimum values about 7th sensor data of the left foot;
Phase 2	Max values about front foot; signals of the right foot; Max values about y-axis center of pressure of the right foot; Moving average about front foot; signals of the right foot; Max values about 8th sensor data of the right foot; Last data of a window from front foot; signals of the right foot; Max values about 7th sensor data of the right foot; Max values about x-axis center of pressure of the right foot; Moving average about 8th sensor data of the right foot; Last data of a window from x-axis center of pressure of the right foot; Max values about x-axis center of pressure of the right foot; Moving average about 1st sensor data of the right foot; Minimum values about rear foot; signals of the right foot; Minimum values about y-axis center of pressure of the right foot;
Phase 3	Moving average about sum of total signals from the right foot; Moving average about front foot; signals of the right foot; Max values about front foot; signals of the right foot; Max values about sum of total signals from the right foot; Minimum values about y-axis center of pressure of the left foot; Moving average about front foot; signals of the left foot; Moving average about 7th sensor data of the right foot; Moving average about 8th sensor data of the right foot; Moving average about y-axis center of pressure of the left foot; Minimum values about front foot; signals of the right foot; Last data of a window from front foot; signals of the right foot; Max values about 6th sensor data of the right foot; Max values about 7th sensor data of the right foot; Max values about 8th sensor data of the right foot;
Phase 4	Max values about x-axis center of pressure of the left foot; Max values about y-axis center of pressure of the left foot; Minimum values about x-axis center of pressure of the left foot; Moving average about x-axis center of pressure of the left foot; Last data of a window about x-axis center of pressure of the left foot; Max values about front foot; signals of the left foot; Last data of a window about y-axis center of pressure of the left foot; Moving average about 9th sensor data of the left foot; Max values about 7th sensor data of the left foot; Max values about 9th sensor data of the left foot; Moving average about y-axis center of pressure of the left foot; Minimum values about 7th sensor data of the left foot; Minimum values about y-axis center of pressure of the left foot; Moving average about 9th sensor data of the left foot; Last data of a window about y-axis center of pressure of the left foot;

**Table 2:** Overall performance of CG prediction models.

Age	Model Name	Correlation Coefficient			RMSE (mm)			rRMSE (%)		
		Anterior/ Posterior	Medial/ Lateral	Proximal/ Distal	Anterior/ Posterior	Medial/ Lateral	Proximal/ Distal	Anterior/ Posterior	Medial/ Lateral	Proximal/ Distal
Young	Model 1 (None)	0.99 (0.99–0.97)	0.91 (0.95–0.71)	0.92 (0.98–0.72)	65.01 ± 3.10	11.97 ± 1.15	9.98 ± 0.59	5.37 ± 0.55 **	25.86 ± 2.16 **	22.03 ± 1.65 **
	Model 2 (RFE)	0.99 (0.99–0.98)	0.89 (0.95–0.66)	0.85 (0.96–0.07)	62.11 ± 8.71	11.94 ± 1.55	11.49 ± 1.44	4.99 ± 0.69**	22.71 ± 1.97 *	26.14 ± 1.87 **
	Model 3 (MI)	0.99 (0.99–0.99)	0.86 (0.89–0.80)	0.92 (0.96–0.72)	33.91 ± 2.57	10.25 ± 1.02	8.96 ± 0.68	3.02 ± 0.20	19.90 ± 1.66	22.16 ± 1.43 **
	Model 4 (ELA)	0.99 (0.99–0.99)	0.87 (0.89–0.80)	0.89 (0.97–0.65)	37.65 ± 2.77	10.24 ± 0.99	9.38 ± 0.70	2.73 ± 0.20	19.52 ± 1.29	21.24 ± 1.23 **
	**Proposed**	**0.99 (0.99–0.99)**	**0.92 (0.98–0.75)**	**0.92 (0.98–0.60)**	**26.73 ± 2.92**	**8.72 ± 1.68**	**6.12 ± 0.72**	**2.13 ± 0.21**	**14.24 ± 1.72**	**14.01 ± 1.09**
Old	Model 1 (None)	0.99 (0.99–0.83)	0.89 (0.98–0.80)	0.85 (0.97–0.63)	84.52 ± 12.93	15.03 ± 0.99	12.41 ± 0.74	7.12 ± 1.06 **	22.92 ± 1.76 *	23.83 ± 1.44 **
	Model 2 (RFE)	0.99 (0.99–0.98)	0.91 (0.99–0.64)	0.89 (0.95–0.63)	59.64 ± 5.14	5.78 ± 1.29	7.1 ± 0.73	4.87 ± 0.43 **	12.71 ± 2.98	16.14 ± 1.11
	Model 3 (MI)	0.99 (0.99–0.98)	0.92 (0.99–0.70)	0.89 (0.98–0.71)	55.03 ± 3.46	5.06 ± 1.10	6.53 ± 0.96	4.48 ± 0.28 *	10.43 ± 2.41	14.37 ± 1.36
	Model 4 (ELA)	0.99 (0.99–0.98)	0.92(0.98–0.67)	0.91 (0.98–0.59)	47.77 ± 4.73	6.20 ± 1.08	6.62 ± 0.83	3.86 ± 0.36	12.72 ± 2.33	14.68 ± 1.28
	**Proposed**	**0.99 (0.99–0.99)**	**0.96 (0.99–0.90)**	**0.91 (0.97–0.79)**	**25.86 ± 2.15**	**5.74 ± 1.08**	**5.39 ± 0.84**	**2.10 ± 0.17**	**11.70 ± 2.42**	**11.74 ± 1.27**

*, significant difference (*p* value < 0.05) between the proposed model and model 1, model 2, model 3, or model 4. **, significant difference (*p* value < 0.01) between the proposed model and model 1, model 2, model 3, or model 4.

**Table 3:** Average measured and predicted CG trajectory PV ranges for age categories.

CG Trajectory PV Range (mm)			
Direction	Age	Test	Prediction
Medial/lateral	Young	45.25 ± 2.53	44.15 ± 2.64
	Old	64.32 ± 2.87	61.35 ± 4.34
Proximal/distal	Young	41.36 ± 2.11	40.09 ± 3.61
	Old	46.84 ± 2.72	45.22 ± 3.01

## Data Availability

Data are available from the authors upon reasonable request.
